# Long‐term spatiotemporal dynamics in a mountain birch (*Betula pubescens* ssp. *czerepanovii*) forest in south‐east Norway

**DOI:** 10.1002/pei3.10087

**Published:** 2022-08-06

**Authors:** Per Holm Nygaard, Fredrik Bøhler, Bernt‐Håvard Øyen, Bjørn Tveite

**Affiliations:** ^1^ Norwegian Institute of Bioeconomy Research, NIBIO Ås Norway; ^2^ AT Skog SA Skien Norway; ^3^ Coastal Forestry, Bredsgården Bryggen Bergen Norway

**Keywords:** *Betula pubescens* ssp. *czerepanovii*, biomass, browsing, disturbance, *Epirrita autumnata*, forest line, permanent plots, stem dynamics

## Abstract

Mountain birch forest covers large areas in Eurasia, and their ecological resilience provides important ecosystem services to human societies. This study describes long‐term stand dynamics based on permanent plots in the upper mountain birch belt in SE Norway. We also present forest line changes over a period of 70 years. Inventories were conducted in 1931, 1953, and 2007. Overall, there were small changes from 1931 up to 1953 followed by a marked increase in biomass and dominant height of mountain birch throughout the period from 1953 to 2007. In addition, the biomass of spruce (*Picea abies*) and the number of plots with spruce present doubled. The high mortality rate of larger birch stems and large recruitment by sprouting since the 1960s reveal recurrent rejuvenation events after the earlier outbreak of the autumnal moth (*Epirrita autumnata*). Our results demonstrate both a high stem turnover in mountain birch and a great ability to recover after disturbances. This trend is interpreted as regrowth after a moth attack, but also long‐term and time‐lagged responses due to slightly improved growth conditions. An advance of the mountain birch forest line by 0.71 m year^−1^ from 1937 to 2007 was documented, resulting in a total reduction of the alpine area by 12%. Most of the changes in the forest line seem to have taken place after 1960. Regarding silviculture methods in mountain birch, a dimension cutting of larger birch trees with a cutting interval of c. 60 years seems to be a sustainable alternative for mimicking natural processes.

## Graphical abstract

The elevation of the mountain birch forest limit at the alpine climate station (1060 m a. s. l.) established in 1932. Left photo from 1960 shows only small patches of mountain birch. Right photo from 2007 shows that the forest line has advanced above the climate station.

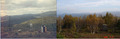

## 1 INTRODUCTION

North‐boreal subalpine forests in Scandinavia and the treeline ecotone dominated by mountain birch (*Betula pubescens* Ehrh ssp. *czerepanovii* [N. I. Orlova] Hämet‐Ahti) have received much attention in the past decade particularly in the Arctic areas (Jepsen et al., 2013; Kuuluvainen et al., 2017; Svensson et al., 2020; Tenow, 1972; Tenow, 1975; Tenow et al., 2004; Tenow & Bylund, 2000; Vindstad et al., 2018). The focus has been on outbreaks of geometrid moths, changes in the treeline, the establishment of Norway spruce (*Picea abies* L*. Karst*.), habitat changes for semi‐domestic reindeer (*Rangifer tarandus* L.), and climate change effects (Tenow, 1972; Tenow, 1975; Aas & Faarlund, 2000; Larsson, 2004; Bryn, 2006; Bryn, 2008; Anschlang et al., 2008; Speed et al., 2010; Van Bogaert et al., 2011; Öberg & Kullman, 2012; Speed et al., 2015; Odland, 2015; Bryn & Potthoff, 2018; Forbes et al., 2020; Holtmeier & Broll, 2019; Stark et al., 2021; Hansson et al., 2021; Kullman, 2021). The observed expansion into the alpine tundra has raised concerns regarding its impact on biodiversity, utilization, landscape aesthetics, and tourism (Bryn & Debella‐Gilo, 2011; Harrison, 2020). Conversely, the expansion has also sparked optimism due to its potential for future carbon storage and increased bioenergy production (Hansen et al., 2021; Ranta et al., 2020; Speed et al., 2015), although concerns about the opposite have also been raised (Hartley et al., 2012). In Norway, mountain birch forest covers about 40,000 km^2^ (Moen, 1998) and constitutes a distinct belt between the upper coniferous forest and the alpine vegetation zone.

The observed expansion of mountain birch forest at landscape scale is claimed to be an effect of changes in both climate and land use, but separating these factors is problematic (Karlsson et al., 2007; Kjällgren & Kullman, 1998; Van Bogaert et al., 2011). In addition, natural disturbances like outbreaks of the autumnal moth (*Epirrita autumnata* Bkh.) and the winter moth (*Operophtera brumata* L.) (Jepsen et al., 2013; Tenow, 1972; Tenow, 1975; Tenow et al., 2005; Tenow & Bylund, 2000; Tenow & Nilssen, 1990), forest fires, avalanches, and extreme weather events temporarily decrease the forest line and the area of the upper subalpine forest belt, the protection belt (Hansson et al., 2021; Holtmeier & Broll, 2019). Anthropogenic disturbances are known to play a major role in the subalpine region. Most of the subalpine areas in southern Norway have been intensively exploited by summer farming since the Iron Age. After the Black Death in the 14th Century, the population decreased, and most dairies were abandoned for 200 years or more (Nordrum & Ødegård, 2004). Summer farming affects the subalpine forest mainly through logging for firewood, coppicing, and intensive grazing which prevents regeneration. Locally, this has been shown to have had a crucial impact on subalpine forest and the treeline during the last 300 years (Bryn, 2008; Bryn & Potthoff, 2018; Hansson et al., 2021; Holtmeier & Broll, 2019; Karlsson et al., 2007; Odland, 2015; Rössler et al., 2008). Large‐scale experimental evidence has to a large extent confirmed these observations (Speed et al., 2015).

Nevertheless, there is little doubt that the growth patterns and distribution of mountain birch forests are sensitive to climate, in particular, the summer temperature, precipitation, and the length of the vegetation period (Holtmeier & Broll, 2019; Kjällgren & Kullman, 1998; Kullman, 2021; Moiseev et al., 2022; Mork, 1968). A short, cold, and moist growing season, and weathering from wind and heavy snowpack is a feature of the harsh environment in mountain birch forests and determines the low growth rates, polycormic crooked growth habits, and short lifespan of stems, which in sum limit the tree height of mountain birch (Tranquillini, 1979). For this reason, dominant height (the mean height of the dominant trees, top height) is used as an index for stature in mountain birch forests (Jonsson, 2004). Jonsson (2004) states that the higher stem turnover at lower tree stature is an effect of higher mortality due to a harsh environment and limited growth resources, resulting in reduced resistance to herbivores and pathogens. Older stems are reported to be weakened and more susceptible to defoliation by the autumnal moth *Epirrita autumnata*, which plays a crucial role in stem dynamics in mountain birch (Tenow, 1972; Tenow, 1975; Tenow et al., 2005; Tenow & Bylund, 2000; Tenow & Nilssen, 1990). Dead or weakened stems are replaced by sprouting from the stump and coarse roots, forming multistemmed individuals. This polycormism is a conspicuous trait of mountain birch (Tenow et al., 2004; Verwijst, 1988).

Understanding the dynamics of mountain birch forests is necessary to predict future changes in biomass, structure, and tree and forest lines but also to foresee responses in mountain plant communities (Felde et al., 2012; Harrison, 2020). Such knowledge is also indispensable when harvesting for bioenergy is considered, especially if one is aiming for a forestry approach mimicking natural dynamics in silviculture (Jacobsen, 2001). Temporal studies of treelines have been conducted using old aerial photographs and maps to detect changes (Bryn, 2008; Kullman, 1991; Tømmervik et al., 2009), whereas studies of biomass and stem dynamics are usually based on reconstructions as measurements from the past are missing (Dalen & Hofgaard, 2005; Hofgaard et al., 2009; Hytteborn et al., 1987; Jonsson, 2004; Kullman, 1991; Kullman, 1995; Kullman, 2004; Moiseev et al., 2022). Stand reconstruction in mountain birch is difficult because stems are relatively short‐lived and are rapidly decomposed after death. Besides, implementing dendrochronological studies on birch is challenging due to the complexity of the growth rings (Levanic & Eggertsson, 2008). This highlights the importance of permanent plots in mountain birch forests measured over many decades. Such empirical data are unfortunately scarce from mountain birch forests. The data set applied in this study covers a time span of nearly 80 years in permanently marked plots close to the sensitive forest line ecosystem from the southern Scandes, the Scandinavian Mountain chain.

The aim of our study was to reveal and document natural forest stand and forest line dynamics in mountain birch forests and further discuss the observed changes in biomass and structure in relation to climate and disturbances. Based on the observed structures and responses, we additionally put forward recommendations about close‐to‐nature management of mountain birch forests which is important for ecosystem services like wildlife and the protection of forests at lower altitudes. Even if the presented study is on a local scale, we think the findings have general application for large parts of the mountain birch forests in the southern Scandes. We hypothesized that repeated attacks of autumnal moth (*E. autumnata*) would cause temporary decreases in biomass production and in tree establishment above the forest line, whereas higher summer temperatures, longer vegetation period, and permanent low herbivore densities would increase biomass production and tree establishment above the forest line.

## 2 METHODS

### 2.1 Study area

**FIGURE 1:**
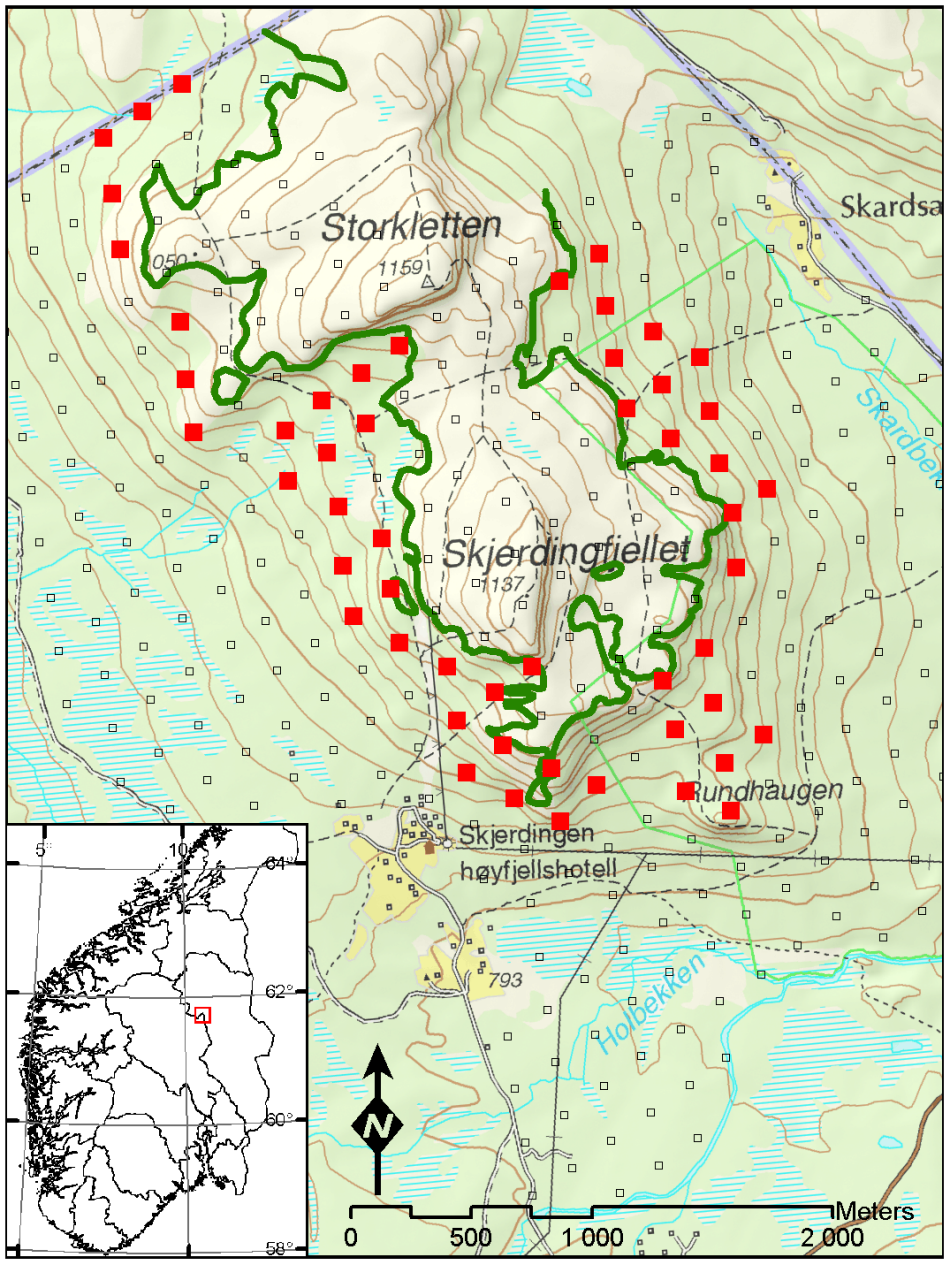
Location of the Hirkjølen experimental area and the 200 × 200 m grid of vegetation plots. The selected 53 plots in the mountain birch belt are shown as red squares. The forest line mapped by Mork and Heiberg (1937) is shown as a dark green line.

The study site is in the Hirkjølen experimental area (61°40′ N, 10°30′ E) in Ringebu, south‐east Norway, between the two major valley systems Gudbrandsdalen and Østerdalen (Figure 1).

The location was selected as a research area for ecological investigations in mountain forests in 1930 and represents the most intensively studied mountainous forests in Norway. The experimental area covers 263 ha of mountain heath and 1153 ha of forest land dominated by mountain birch and Norway spruce with a minor component of Scots pine (*Pinus sylvestris* L.) (Mork & Heiberg, 1937). The elevation ranges from 740 to 1160 m a.s.l. The bedrock consists of dark sparagmite with some occurrences of phyllite layers and limestone. The dominant soil type is iron podzol, but a minor proportion is classified as brown soil (Semb, 1937).

**FIGURE 2:**
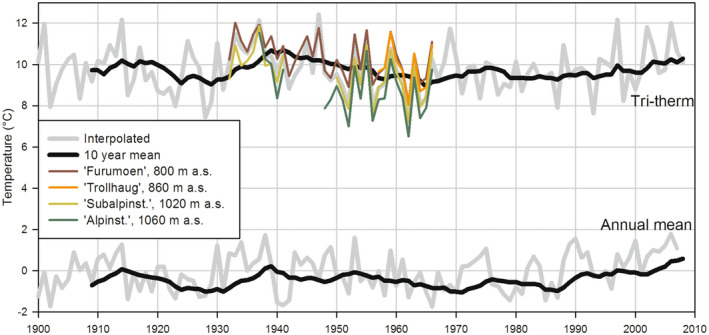
Chronology of tri‐term (mean temperature for Jun–Aug) and annual mean temperature. The gray line shows the interpolated chronology for mean latitude, longitude, and elevation (947 m.a.s.l.) of the selected sample plots. The black line shows the mean of the preceding 10 years. In addition, the tri‐term chronologies from four of Mork's (1968) climate stations located inside the experimental area are plotted.

The local climate is shown in Figure 2, based on data from four of the local climate stations setup in the study area and an interpolated climate chronology. The interpolated chronology spanning from 1900 to 2008 is based on measurements from the Norwegian Meteorological Institute. The chronology was constructed with a polynomial regression of elevation, latitude, and longitude using data from the nearest climate stations within ±1.25° latitude and ±2.5° longitude (Ojansuu & Henttonen, 1983). Mean latitude, longitude, and elevation of the field plots were used as input values for the interpolated temperature series plotted in Figure 2. When the same interpolation model was used for precipitation, the mean annual precipitation in the period 1931–2007 was 645 mm.

The summer dairy history at Hirkjølen state common land (9500 ha) began during the 1600s. With a growing population, the farming increased and reached a maximum around 1850. Figures from the Norwegian cadastre protocols in 1723 showed that the number of cattle at Hirkjølen was 220. The number of cattle increased to 569 in 1845 when the browsing reached a maximum. Also, pollen analysis from the area indicates a maximum of browsing around 1850 (Høeg, 1994). At the beginning of the study period (1930), there were 19 active summer dairy farms close (1–6 km distance) to the experimental area, which kept in total ~ 270 cattle and some sheep. The grazing intensity was described as high and was reported to suppress birch regeneration at a lower altitudinal range limit, but the effect diminished with increasing altitude and distance from the dairy farms (Mork, 1947; Mork, 1958; Mork, 1967; Mork & Heiberg, 1937). Mork (1948) claimed that there were no effects of cultural activities on the forest's upper line which was determined by climate. The cattle did not graze that high up, sheep were rarely kept, outfield pasturing with goats was prohibited, and firewood was not collected at that high elevation. A recent mapping of grazing conditions in the Hirkjølen area also displays low quality for grazing above the forest limit (Rekdal, 2013). Today the number of cattle has decreased to c. 20. However, increased populations of reindeer and moose after 1970 have probably increased the browsing of birch in the area (Austrheim et al., 2008).

Since 1930, several silvicultural experiments and commercial loggings were conducted in the spruce‐dominated hillsides. The belt‐cuttings from 1951 to 1965 and the later commercial logging of Norway spruce stopped well below the altitude of the presented plots. Because of previous overexploitation of the lower altitudinal range of the mountainous forest in Norway, a protection zone was established by law in 1908 which prohibited cuttings at this altitude. Our study is carried out in the upper part of this protection belt, spanning the altitude gradient from 850 to 1050 m a.s.l. We emphasize that the mountain birch plots in our study show very little or no influence by man over the last 80 years and little anthropogenic influence during the last 150 years.

**FIGURE 3:**
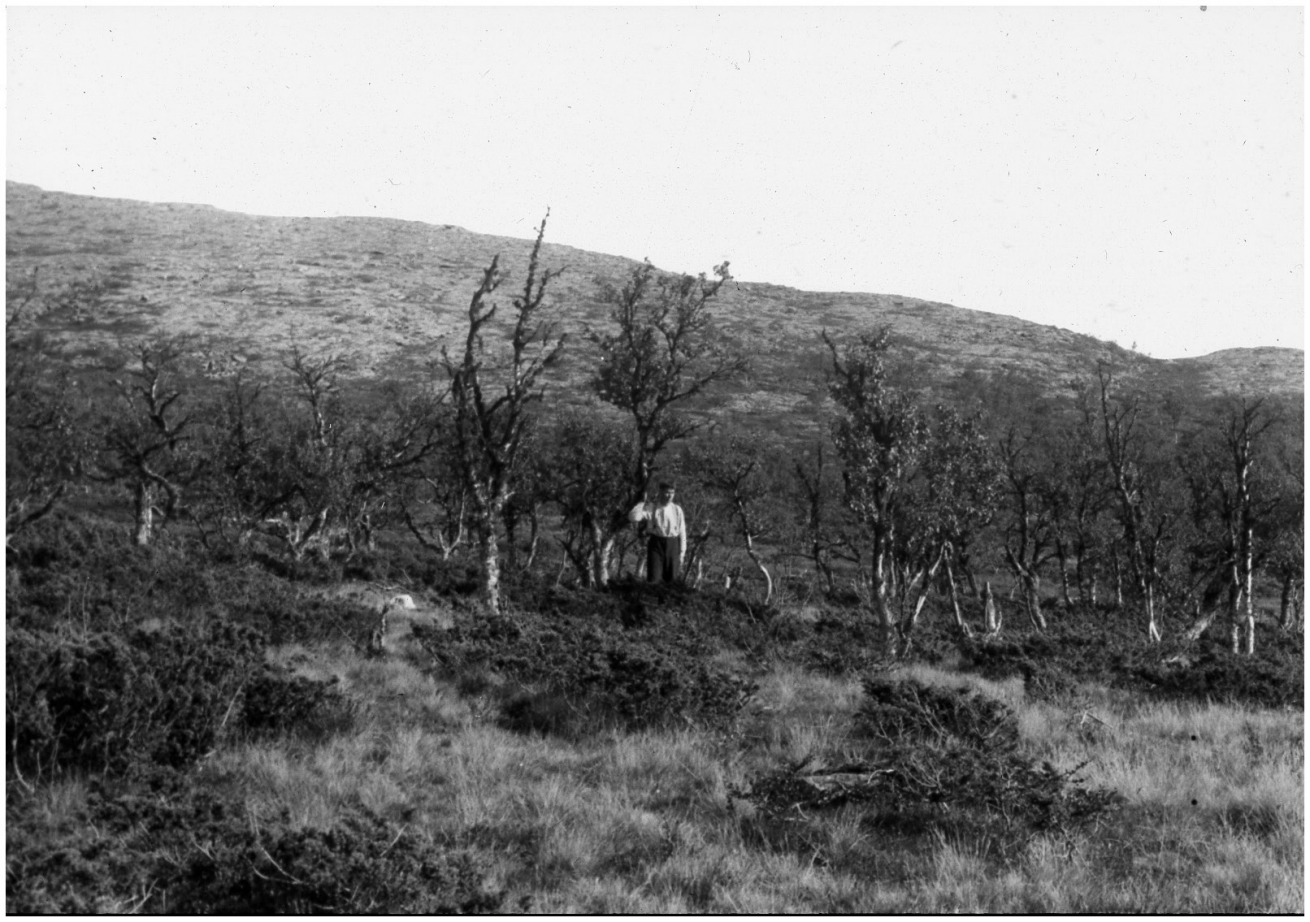
Trees in the Hirkjølen experimental area were severely attacked by a moth (*Epirrita autumnata*) (Mork & Heiberg, 1937).

Among natural disturbances, geometrid outbreak is probably the main driver for mountain birch forest dynamics (Vindstad et al., 2018). A thorough review of outbreaks in Scandinavia from 1862 to 1968 is given by Tenow (1972). Based on qualitative and quantitative observations, the Hirkjølen experimental area seems to be influenced by the autumn moth (*Epirrita autumnata*) in 1862, 1891,1919,1930,1948,1955, 1965, 1975, and 1992 (Tenow, 1972, Forest Director 1976, 1986, and pers. comm.). However, the precision of the outbreak localities is varying and only a few sources are mentioning Hirkjølen by name. In general, the observations refer to the main valley, district, or region, and little information is presented on the strength of the attacks. In Tenow (1972), an outbreak at Hirkjølen is mentioned with a question mark to Mork (1968), but the same observation of Mork is published as a photo as early as 1937 showing conspicuous damage to birch trees (Figure 3).

Mork and Heiberg (1937) are claiming that the mountain birch on the photo was damaged some years ago, and remarks in the field dairy show 1934. Unpublished data from the forest inventory in 1953 also confirm an outbreak within the research area some years ago, probably in 1948.

### 2.2 Sampling

The mountain birch forest line in the experimental area was accurately mapped in 1934–1935 by theodolite and triangulation work based on the definition of Mork, defining the upper limit as a forest with spacing less than 30 m between trees higher than 3 m (Mork & Heiberg, 1937). The forest line was published on the vegetation map from the Hirkjølen experimental area which is assumed to be the first vegetation map from Norway. A remapping of the forest line was carried out in 2005–2007 following Mork's criteria. The forest line was mapped by a combination of field measurements of tree height, tree distances, GPS measurements, and use of laser scanning. The laser data acquisition was carried out 26th of June in 2005. A fixed‐wing Piper PA31 Navajo aircraft was used with an Optech ALTM‐3100 laser scanner. This resulted in a point density on the ground of approximately 3.4 m^−2^. Data were initially processed by the contractor (Blom Geomatics, Norway). More detailed information about laser scanning is given by Bollandsås et al. (2013). Tree height of mountain birch was calculated as the difference between max height and ground level and every tree higher than 3 m was positioned in GIS (Appendix S1 and S2).

A systematic triangle design of 347 plots (10 × 10m) was established in 1931 for vegetation and forest inventories (Figure 1). Two hundred and seventy‐six plots were located below the forest line. From these plots, we selected 53 plots located within the protection zone of the mountain birch belt (Figure 1). The selection criteria were

- that the plots were located above the elevation of the later experimental loggings and
- that the plots were measured in all three inventories, i.e. located below the forest line mapped by Mork and Heiberg (1937).

These 53 plots span a poor‐rich vegetation gradient from Vaccinium forest dominated by *Vaccinium myrtillus* (L.), *Avenella flexuosa* (L.), *Melampyrum pratense* (L.), *Trientalis europaea* (L.) to Geranium Forest dominated by *Geranium sylvaticum* (L.), *Aconitum lycotonum* (L.), and *Geum rivale* (L.). The gradient consists of 30 plots belonging to the Vaccinium‐Betula sociation and 23 plots to the Geranium‐Betula sociation (Mork & Heiberg, 1937). The 53 plots also represent a gradient in soil fertility from iron podzol to brown soil profiles.

**FIGURE 4:**
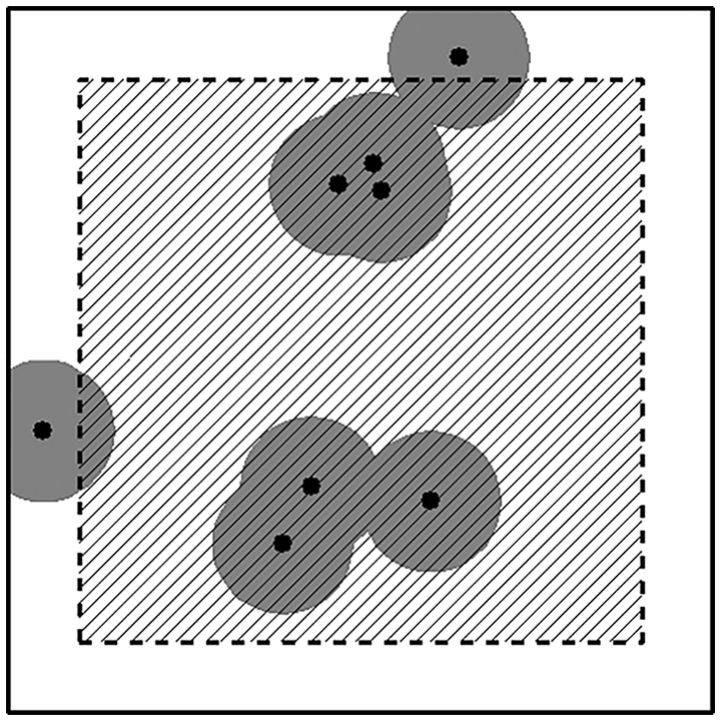
The “occupied area” is defined as the percentage of the plot area (hatched area) covered by the area within a 1 m radius of standing birch stems (gray area) after excluding a 1 m buffer from the plot edge.

Within these 53 plots, the height and diameter at breast height (dbh) of all stems with dbh > 2.5 cm were recorded. For conifers, height was recorded for all stems taller than 0.1 m. Spatial position within the plot was recorded for all registered stems. The same plots were reanalyzed in 1953 and 2007 by the same methods. The retrieval of the plots and the single trees in 2007 was work‐demanding but made feasible by looking for the old plot marks and numbered aluminum tags labeling the trees. Not all tags were retrieved, and in those cases, the previously registered stems were located and identified by using the registered spatial position. Dbh measurements were obtained by using calipers and height measurements by using a measuring rod and theodolite, or by a Vertex hypsometer in the last inventory. A few individuals of Scots pine (*Pinus sylvestris*), rowan (*Sorbus aucuparia* L.), aspen (*Populus tremula* L.), and goat willow (*Salix caprea* L.) were present in the study area but were excluded from the analysis. In the registrations, only stems were identified and not genetic individuals of birch. In contrast to sexual recruitment, vegetative recruitment is limited to the immediate proximity to the mother tree forming multistemmed individuals. Thus, the number and recruitment of stems could be spatially clustered and do not necessarily reflect the occupation of growth space. We therefore used the spatial data to describe the occupation of growth space by quantifying the area covered by a constant buffer around all stems. The area outside this buffer, here denotes as “unoccupied area,” represents the area that is not available for vegetative recruitment. The area covered by this buffer is denoted as “occupied area” and is expressed as a percentage of the total area. The width of this buffer is here set to a constant of 1 m based on our field experience and Frivold (1994) and Verwijst (1988) who state that vegetative recruitment only occurs as sprouts emerging from the stump and coarse roots close to the stem. Besides, we carried out a sensitivity test showing that doubling the buffer had almost no effect on the density of recruited stems. Due to a lack of information on standing trees outside the plot, a 1 m buffer zone inside the plot was excluded from the spatial analysis (Figure 4).

### 2.3 Statistical analysis

We applied the biomass function of Bollandsås et al. (2009) for the above‐ground biomass of mountain birch which is partly based on sample trees from the Hirkjølen experimental area. The Swedish biomass function of Marklund (1988) was used for spruce. Hereafter, the above‐ground biomass of trees is simply referred to as biomass.

To make the mortality in the two periods with different lengths comparable, we calculated a mean annual mortality rate (*m*) from the total mortality in the period (M) assuming mortality of a constant fraction each year in the period, using the following equation (Sheil & May, 1996):

$m=1–{\left(1–M\right)}^{1/t}$,

where $M=1–N_{t}/N_{0}$, where N_0_ is the number of stems at the start of the period, N_*t*_ is the number of stems surviving to the end of the period, and *t* is the number of years in the period. Mortality was also calculated as a fraction of biomass by exchanging a number of trees with biomass in the equation above. Confidence intervals for the annual mortality rates were calculated by converting the confidence limits of the total mortality in the period into annual rates by using the equation above.

The spatial analysis within the permanent plots and positioning of forest lines was carried out using ESRI ArcGIS 9 software.

Dominant height, here calculated as the maximum height of birch at each 100 m^2^ plot, was included as a stand variable to reflect tree stature (Jonsson, 2004). We used paired *t* tests to detect significant changes in the two periods in stem density, biomass, occupied area, and dominant height. Fisher's test was applied to compare mortality rates of young and old stems in the same period. Pearson's correlation coefficient was calculated between biomass, stem density, mean tree size for the different years of observations and altitude, and horizontal and vertical distance to the forest line. Procedures in SAS, release 8.02 (SAS, 1999), were applied for statistical analyses and data treatment.

## 3 RESULTS

**FIGURE 5:**
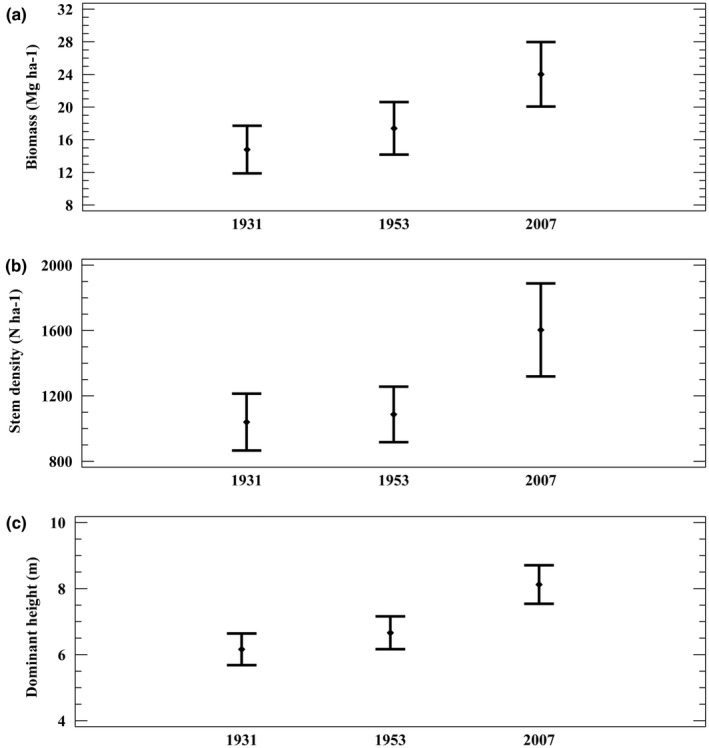
Means and 95% confidence intervals for (a) biomass (Mg ha^−1^), (b) stem density (N ha^−1^), and (c) dominant height (m).

There was a significant increase in biomass from the first inventory to the second and third (Figure 5a). The density of birch stems did not change significantly from 1931 to 1953 but increased by 60% in the second period (Figure 5b). The dominant height of the birch also increased significantly in both periods (Figure 5c). The mean annual increase in dominant height was 2.3 and 2.4 cm year^−1^ for the first and second periods, respectively.

The mean annual increase in biomass shows a similar pattern over the two periods, with 5.0 and 8.14 Mg ha^−1^, respectively (Table 1). Also, the increase in the numbers of standing stems was larger in the second compared with the first period. Mortality and in particular recruitment were higher in the second period (Table 1).

**TABLE 1:** Dynamics of stems and biomass in the two periods with calculated mean annual rates of mortality and growth. Stem density and biomass ha^−1^ (Aas & Faarlund, 2000) are the mean values of the 53 plots. Rates for the whole period and annual rates are calculated on the basis of all birch trees. Growth rates are calculated for the surviving stems only

	1931–1953					1953–2007			
	Mean	±SD	Total rate in the period (%)	Calculated annual rate (% year^−1^)		Mean	±SD	Total rate in the period (%)	Calculated annual rate (% year^−1^)
Stem density									
Mortality (N ha^−1^)	260	213	25	1.30		636	444	58	1.61
Recruitment (N ha^−1^)	309	230				1151	916		
Biomass									
Mortality (Mg ha^−1^)	3.62	4.84	24	1.27		11.05	8.94	63	1.85
Growth (Mg ha^−1^)	5.00	3.52	45	1.69		8.14	6.23	128	1.54
Recruitment (Mg ha^−1^)	1.23	0.99				8.74	8.23		

**FIGURE 6:**
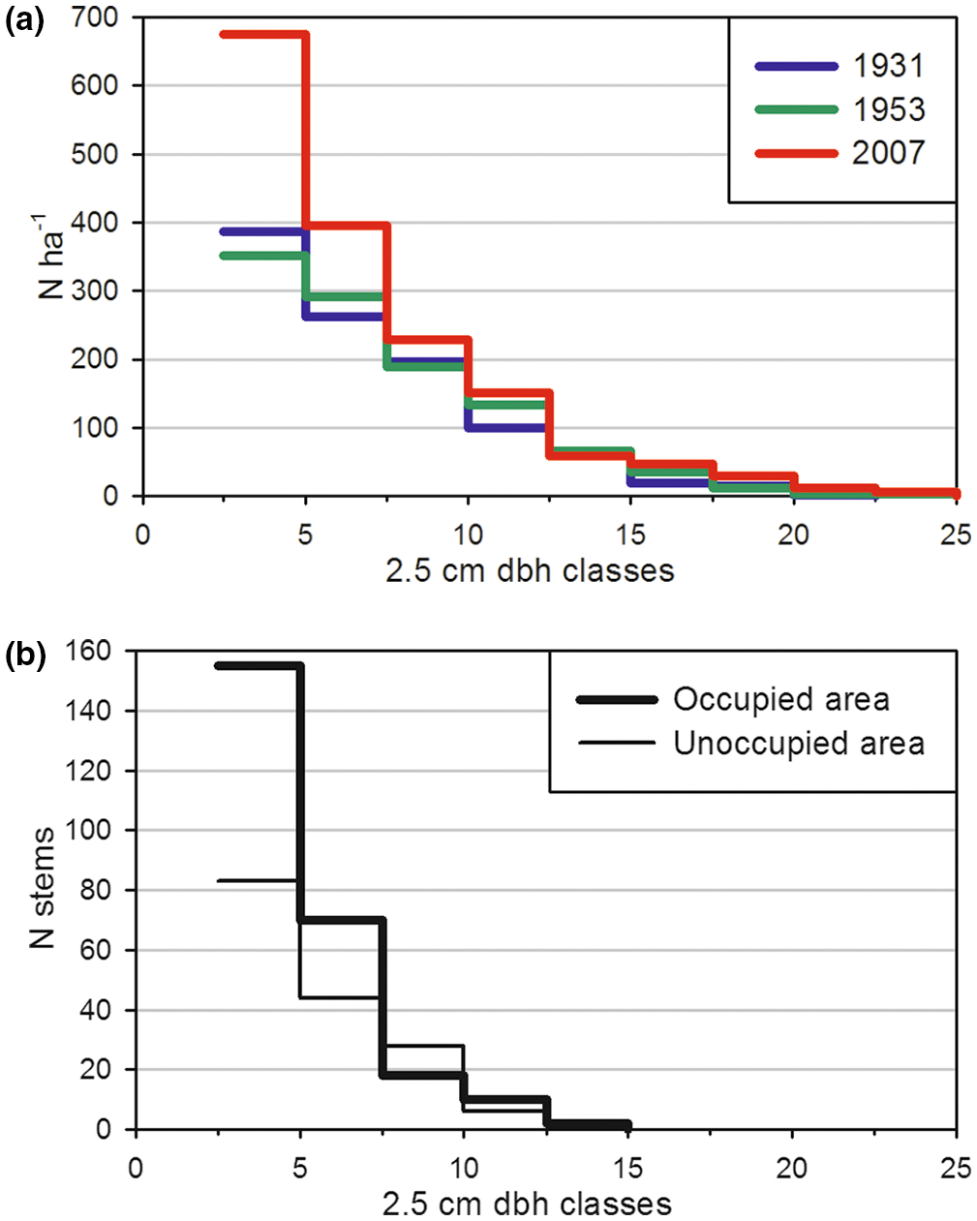
(a) Mean density of birch stems grouped into 2.5 cm dbh classes for 1931, 1953, and 2007. Stems <2.5 cm were not registered. (b) The total number of birch stems in 2007 recruited after 1953 was grouped into 2.5 cm dbh classes in the occupied and unoccupied areas.

There was a slight increase in the number of large stems (dbh >9 cm) in the first period, whereas, in the second period, there was a significant increase in the number of small stems (Figure 6). However, the percentage of occupied area increased only slightly (18.8% in 1931, 19.7% in 1953, and 21.4% in 2007) and not significantly over the two periods.

**FIGURE 7:**
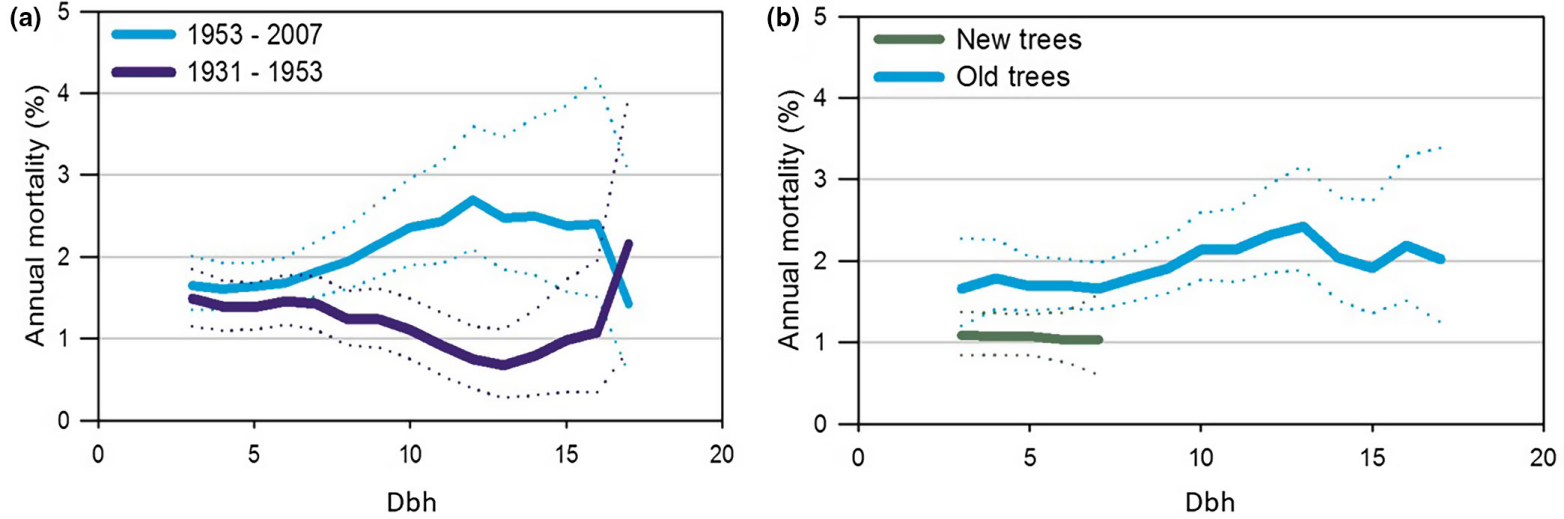
(a) Mean annual mortality with 95% confidence interval in a 5 cm floating interval of dbh measured in 1931 and dbh measured in 1953 and (b) Mean annual mortality with 95% confidence interval in a 5 cm floating interval of old trees dbh measured in 1931 and 1953–2007 and new trees dbh measured in 1953 for the period 1953–2007.

A total of 27% of the stems standing in 1931 persisted until 2007. These stems contributed to 73% of the birch biomass standing in 2007. Among the stems registered in 1931, the small stems (dbh < 7.5 cm in 1931) had a similar mean annual mortality rate in the two periods. In contrast, the larger stems had a lower mortality rate than the smaller stems in the first period whereas a higher rate in the second period (Figure 7). Stems recruited after 1931 had a lower mortality rate (45%) than older stems (64%) in the period 1953–2007 (F‐test: *p* < .0001) (Figure 7b). Mortality was not correlated to density or biomass in any of the two periods.

The recruitment of birch was strongly aggregated around old stems. In total, 61% of the stems recruited after 1953 were found within the occupied area, which covered only 20% of the total area. The mean diameter of recruited stems was slightly smaller (*t* test, *p* = .026) in the occupied area, with a diameter distribution skewed more to the left (skewness = 1.5) compared with recruitment in the unoccupied area (skewness = 0.9) (Figure 6b). Stems recruited in the period 1931–1953 had a similar proportion located in the occupied area (63%).

**FIGURE 8:**
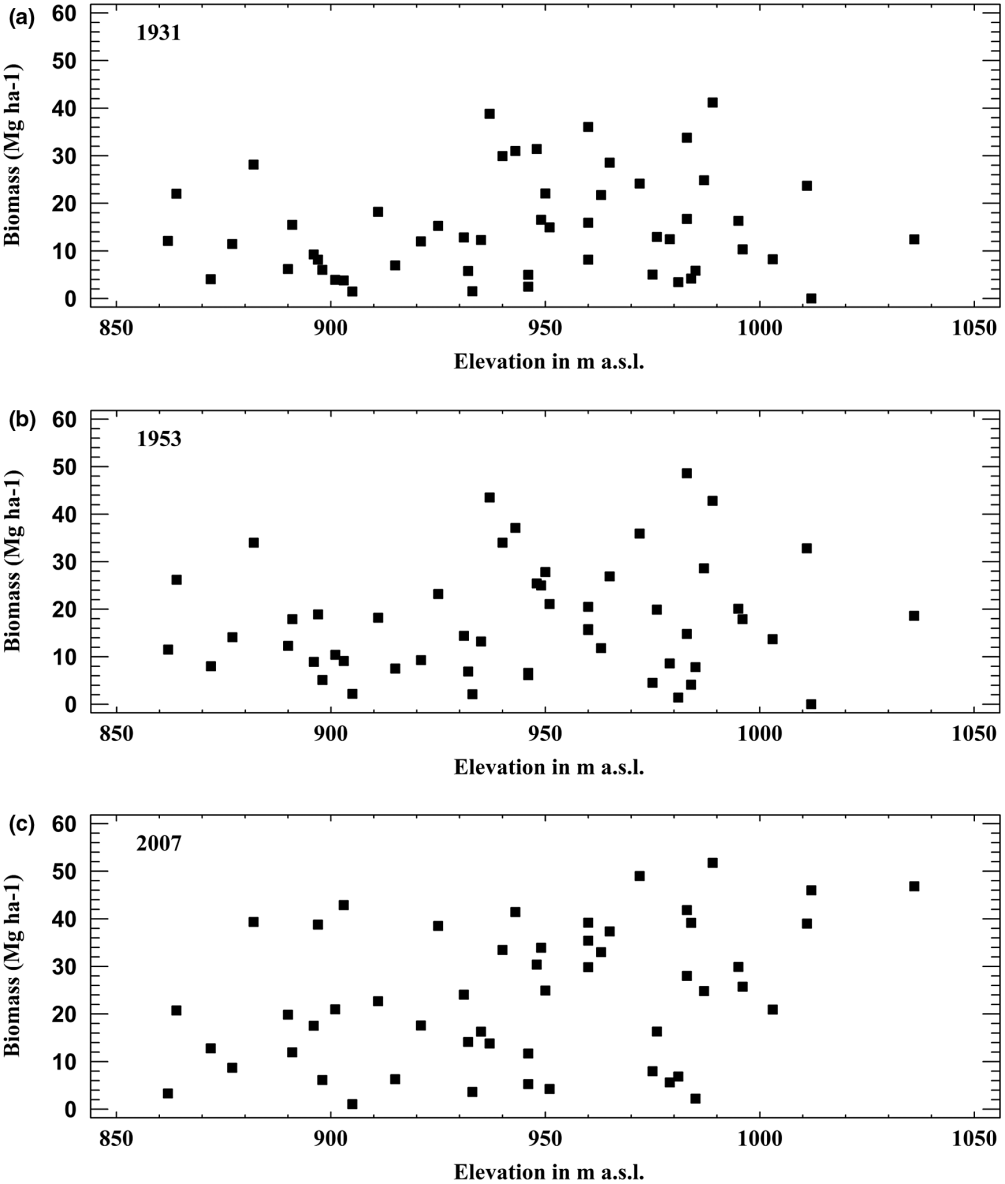
Distribution of birch biomass (Mg ha^−1^) by elevation for (a) 1931, (b) 1953, and (c) 2007.

Birch biomass by elevation for the different years is shown in Figure 8a–c.

Elevation or distance to the reference forest line mapped in 1937 and the measured or calculated changes in forest attributes on the plots showed no clear relationship. However, the number of recruited stems per plot in the unoccupied area in the last period (Figure 6b) was positively correlated to elevation (r = 0.44, *p* < .01). In the 2007 inventory, elevation was positively correlated with both occupied area (r = 0.40, *p* < .01), stand density (r = 0.38, *p* < .01), and biomass of birch (r = 0.37, *p* < .01). The correlation indicates relatively weak relationships between the variables. The shift in biomass is mostly due to the substantial birch growth in the four plots above 1000 m (Figure 8c).

Of the 22 plots where spruce was present in 2007, the first establishment of spruce had occurred in six plots after 1953 and in two plots in the period 1931–1953. In three plots, spruce was only represented by seedlings in 1931. The spruce biomass was rather a stable showing 5.5 Mg ha^−1^ in 1931 and 5.6 Mg ha^−1^ in 1953 and it increased to 13.5 Mg ha^−1^ in 2007. Most of the spruce was found in plots below 950 m a.s.l., but a few individuals were also present in the highest elevation plots. The total number of spruce stems (h > 1.3 m) in all plots was 38, 42, and 68 in 1931, 1953, and 2007, respectively. Both sexual and vegetative regeneration had taken place and the number of ramets and genets had increased proportionally (c.1.8 ramets per genet).

**FIGURE 9:**
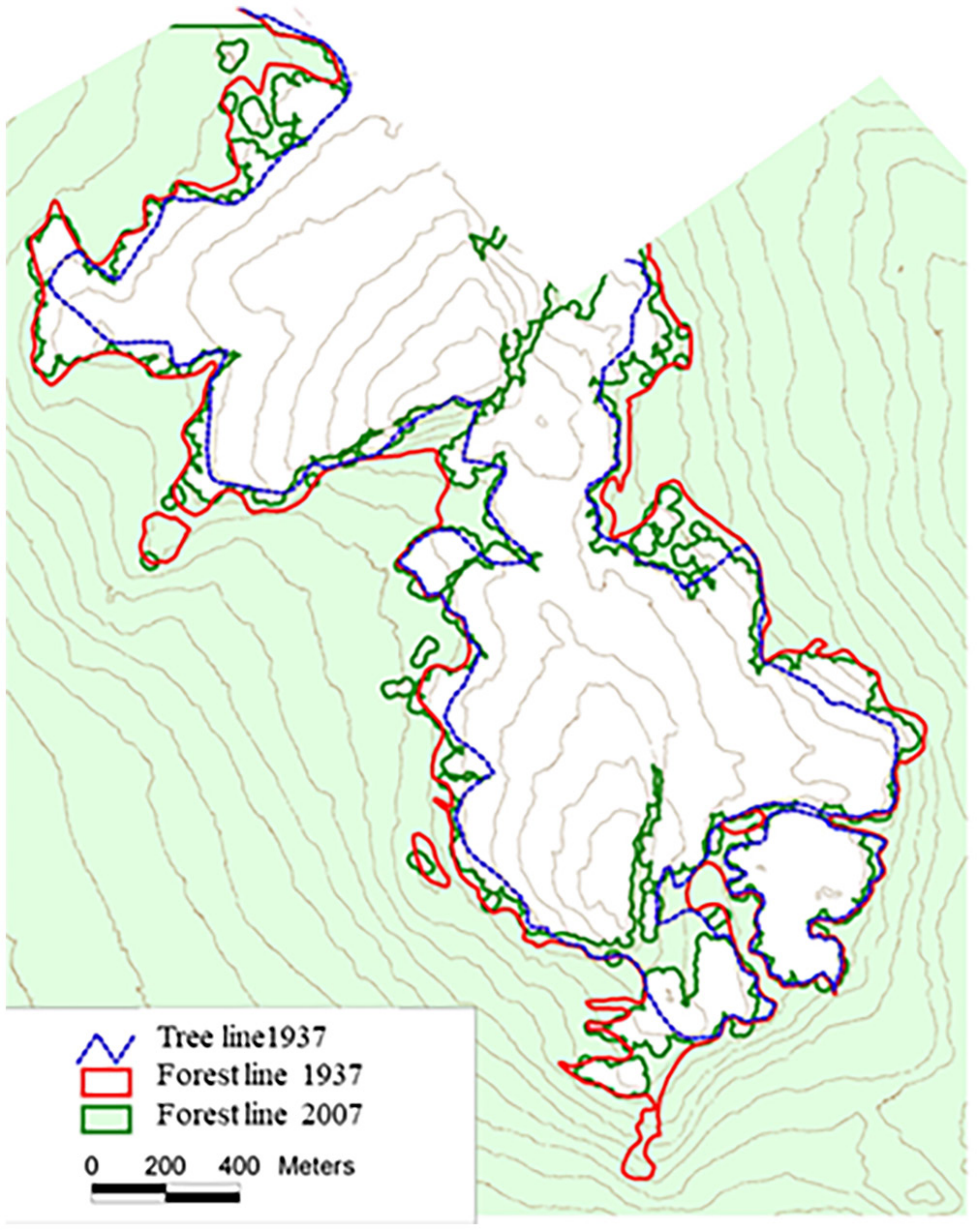
Map showing tree line and forest line from 1937 and the new forest line from 2007.

**FIGURE 10:**
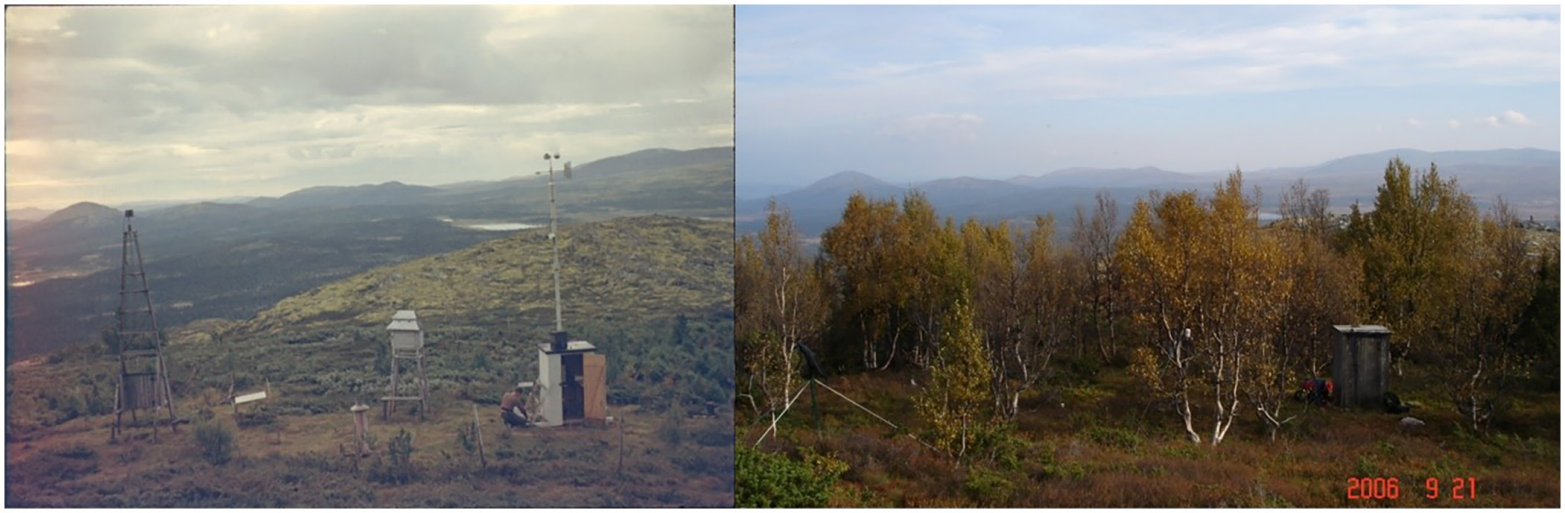
Photographs taken from the same position of the alpine climate station established 5 m above the forest line (1060 m a. s.l.) in 1932. The left photo shows the climate station in 1960, where only small patches of mountain birch can be seen (Photo K. Bjor). To the right is the climate station in 2007, where the forest line has advanced above the climate station (Photo B. Tveite).

The mountain birch forest line in 1937 compared with the forest line in 2007 show a maximum increase in elevation of 50 m, except for regrowth after a destructive avalanche in the southernmost area (Figure 9). A visual inspection showed changes in altitude varied by soil depth, aspect, and local topography, and the highest increase was found in western hillsides particularly in depressions by 0.71 m year^−1^. The area above the forest line was reduced by 12% in the experimental area over the 70 years. The advance in the forest line has taken place over a period of 70 years, but we have no measurements within the period. However, earlier photographs from the alpine climate station established 5 m above the forest line in 1932 show substantial changes from 1960 to 2007 (Figure 10).

## 4 DISCUSSION

### 4.1 General trends

The mean biomass levels in our study (Table 1) are lying within the range of results from other studies in mountain birch forests which span from 9.6 Mg ha^−1^ (Bylund & Nordell, 2001) to 21.2 Mg ha^−1^ (Starr et al., 1998), 27.5 Mg ha^−1^ (Dahlberg et al., 2004), and 35 Mg ha^−1^ (de Wit et al., 2014). Several studies have indicated or shown increases in biomass in Fennoscandian mountain birch forests in recent decades (Hallinger et al., 2010; Hedenas et al., 2011; Rundqvist et al., 2011; Speed et al., 2015; Stark et al., 2021; Tømmervik et al., 2009; Tømmervik et al., 2019), but to our knowledge, this study is among the few to document an increase over 80 years in permanent plots, where growth, ingrowth, and mortality is quantified. For the last period, a higher mortality rate was observed in the larger and older stems compared with the smaller stems. This was not the case for the first period. Further, the presence of a cohort of new stems contribute significantly to the total biomass in 2007. As 61% of the recruited stems were located closer than 1 m to previously registered stems, we regard sprouting as the most important contributor to the increased density and biomass. This indicates that a rejuvenation event took place after the inventory in 1953. As described above, severe moth attacks at Hirkjølen experimental area have been documented nine times for the period 1862 to 1992. On the landscape scale, the attacks recur at intervals on average of 9–10 years (Tenow, 1972). The attacks seem to be cyclic, but which mechanisms lie behind the recurrency is still poorly understood (Tenow et al., 2007, Tenow, 2013), although delayed density‐dependent parasitism is regarded as an important factor for autumnal moth cycles (Tenow et al., 2013). Weather conditions probably play a major role in attacks (Tenow, 1972). Low summer temperatures may predispose mountain birch for attacks because of decreased assimilation (Mork, 1968), but climate warming is also predicted to increase herbivore insect populations (Silfver et al., 2020). The notes to the forest inventory in 1953 (Peder Braathe 1953, unpublished note) state that the most striking result is the high mortality of old mountain birch particularly at higher altitudes. More than a 50% reduction in stem volume growth was recorded, for larger stems (dbh > 10 cm). This statement and evidence fit with Mork's (1968) indications of an apparent decline in the mountain birch forest in the area up to the early 1960s. The increase in elevation for the mountain birch forest line may partly be explained by earlier moth attacks which decreased the forest line and former seedling establishment in warmer periods prior to the new global change. It should also be kept in mind that the recorded forest line is highly dependent on Mork's criteria, meaning that trees with heights lower than 3 m in 1937 have exceeded this limit and reduced the distance to neighbor trees. The increase in elevation is in the same order as reported in other investigations in south‐east Norway (Aas & Faarlund, 2000; Bryn & Potthoff, 2018). A reduction of the alpine vegetation area of 12% will have great implications for biodiversity and ecosystem functioning.

Other authors are also reporting a high proportion of stems originating from sprouting elsewhere in Scandinavian mountain birch forests in the relatively cold period between the 1950s and the 1980s (Dalen & Hofgaard, 2005; Kullman, 1991; Tenow et al., 2004). This suggests that the biomass had a temporary dip after 1953 and a subsequent recovery until the inventory in 2007 that was more rapid than the increase preceding 1953. Hence, this rapid biomass increase should be seen in relation to the rejuvenation event and the subsequent high proportion of young stems with a high relative growth rate. We emphasize that the ability of mountain birch to recover rapidly after moth attacks (Karlsson et al., 2004) should not be misinterpreted as an instant response to climate. Instead, higher summer temperatures, longer vegetation periods, and facilitation probably speed up this recovery.

### 4.2 Mortality

Mean annual mortality for stems smaller than 7.5 cm dbh in 1931 was similar in the two periods, whereas the mortality for larger stems increased significantly from the first period to the last one. We assume that for these stems, the time required to reach 7.5 cm dbh was a minimum of 30 years, considering the maximum size of the new stems in 1953. Hence, it was the stems older than c. 50 years in 1953 that contributed to the high mortality in the 1950s and thereafter (Figure 7a,b). If the above‐mentioned moth outbreaks in 1950s and 1960s are taken into consideration, this fits very well with previous studies that have shown a strong increase in susceptibility to moth defoliation when the age of birch stems approaches c. 60 years (Bylund, 1997; Meyer et al., 2021; Ruohomaki et al., 1997).

Jonsson (2004) reported a life expectancy decreasing from 56 years at stem initiation to less than 10 years by 80–90 years of age in stands with tree stature similar to those in our study. The size–mortality relationship differs in the two periods, which indicates that such age–mortality relationships both depend on the time period studied and the occurrence and strength of the disturbance events within it. This should be considered when using static survivorship curves and retrospective studies of mortality in mountain birch (Hytteborn et al., 1987).

The high mortality of large stems and the substantial contribution of young stems to the biomass in 2007 demonstrate a high biomass turnover. This is an interesting feature when considering a close‐to‐nature approach to the management of mountain birch forests for bioenergy production (Hansen et al., 2021; Jacobsen, 2001). Our results (Figure 7b) suggest that a great proportion of the biomass could have been harvested in 1953 with little or no effect on the standing biomass in 2007, as many of the large stems died in the period anyway. Cutting of these large stems would probably have stimulated even greater sprouting (Vindstad et al., 2015).

### 4.3 Recruitment of mountain birch

We observed substantial recruitment in the last period in the unoccupied area which is assumed to originate from seedlings. This was especially the case for plots at the highest elevations. It is problematic to estimate the exact timing of this due to the long‐time span between the last two inventories and the time required for stems to reach the minimum registration size (2.5 cm dbh). However, the diameter distribution of recruited stems gives no indication of a more recent origin of those recruited in “unoccupied area” than those recruited in “occupied area” (Figure 6a). Although sprouting can be frequent in a cold period, seedling establishment and survival have been shown to be facilitated by warm summers (Hofgaard et al., 2009; Sveinbjörnson et al., 1996; Weih & Karlsson, 1999). A peak in mountain birch seedling establishment in the 1940s has been observed elsewhere in the Scandes (Dalen & Hofgaard, 2005). The seedlings colonizing new space might have originated from the warm period 1930–1950, but these were too small to be registered in 1953. Hence, we consider that the colonization of new growth space at high elevations observed in our study is not a new phenomenon but has been in progress since the 1950s and onward.

We consider that the uppermost part of the mountain birch belt with the lowest tree stature is not well represented in our study by only four plots above 1000 m. This is indicated by both the density, occupied area, and biomass being positively correlated to elevation, in the last inventory. The observed increase in elevation of the mountain birch forest line also shows that high regrowth had taken place in the belt between the old forest line and the tree line. We do not know exactly when the elevation in forest line took place during the period, but old photographs show that large increases occurred after 1960, which concur very well with the observed biomass accumulation. For future permanent plot studies of long‐term dynamics in mountain forests, we therefore recommend coverage of a greater altitudinal gradient. Unfortunately, this was not possible in our study because plots above the forest line were not analyzed previously.

### 4.4 Recruitment of spruce

The number of plots with spruce present in the study area has doubled during the last century. Plots with the largest biomass of spruce were mostly at lower elevations due to the natural zonation of birch and spruce. However, spruce also proves to be able to establish single trees and groups at sites to the upper forest line, a similar pattern which is observed in other mountain areas in Norway (Øyen & Nygaard, 2020). Small dwarf individuals of spruce are shown to use 60–100 years for reaching dbh in subalpine forests (Nygaard & Stuanes, 2018). Judging from the size of the new genetic individuals in 2007, neither the colonization of new plots nor the establishment of new spruce seedlings seems to be restricted to the recent period of climate warming. Mork (1958) concluded from his fencing experiments in Hirkjølen that the absence of naturally established spruce seedlings at the highest elevation plots was caused by a lack of viable seeds and not by grazing livestock. After the rich seed year in 1934 only, two rich flowering years for spruce were observed from 1936 to 1966, but both these summers were too cold for ripening spruce seeds (Mork, 1967; Mork, 1968). The production of viable spruce seeds depends on warm summers (Mork, 1968), suggesting that a warmer summer climate will increase the potential for spruce colonization in mountain birch forests in the future.

### 4.5 Changes relating to local climate and land use

Recent climate warming does not satisfactorily explain the observed biomass increment as the summers preceding 2007 were actually colder than those preceding 1953 (Figure 2). In addition, the observed changes as described here seemed to have started prior to the period of recent climate warming.

One hypothetical causal factor is the suppression of sprouts and seedlings by grazing livestock up to WW2 when pastoral farming declined. Young sprouts from birch are preferred fodder for ungulates like cattle, reindeer, and sheep (Bryn & Daugstad, 2001; Lehtonen & Heikkinen, 1995; Nedkvitne et al., 1995; Speed et al., 2010; Tenow et al., 2005). However, if Mork (1948) is correct in assuming that cattle did not graze at the elevation of the forest line and that sheep were rarely kept, this cannot explain the observed changes in the uppermost plots. It is shown that even low densities of ungulates can depress the treeline below its potential by limiting the establishment of new tree recruits (Speed et al., 2010), but to our knowledge, the number of sheep at Hirkjølen in the last 70 years has been just a few for household purpose. Seasonal timing of browsing is shown to be crucial for the effect on vegetation (Stark et al., 2021). A slightly increasing population of wild reindeer are passing through the experimental area from winter to summer range, but the experimental area is not included in their main summer range. The increased population of moose from 1970 and onward are representing a potential increase in summer season browsing. This has probably reduced the regeneration of mountain birch in the Hirkjølen experimental area, but experimental exclusion of browsing is needed to quantify this effect.

Furthermore, Kullman (1991) observed similar changes in a mountain birch forest in northern Sweden where the historical anthropogenic utilization was stated to be very low. He showed that most of the present stems originated from sprouting after 1930, with a peak in sprouting frequency in the 1960s, and stems older than this mainly originated from the 1880s and 1890s. He also stresses that almost all present genets constituting the forest were established prior to the 20th century and that the genets have become increasingly polycormic during the last century. Based on these findings, the increased stem density cannot merely be explained by the secession of grazing. According to Speed et al. (2010) grazing has the potential to limit the establishment of new tree recruits rather than decreasing the survival of existing individuals. The general enhanced forest line supports that recent browsing is not the main driver for the increase in biomass.

As mentioned, sprouting frequency relates both to age and the occurrence of disturbance events. This brings in both time lag and stochasticity, which makes it difficult to relate the observed changes to specific changes in either climate or land use. It also means that climate and land‐use history prior to the study period is relevant. Long‐term effects of previous intensive land use have also been indicated elsewhere in Scandinavia (Östlund et al., 2015; Tømmervik et al., 2019). The fact that the lifespan of genets expands the lifespan of single stems in polycormic birch, necessitates considering timescales exceeding the life span of single stems. Dynamics on a genet level are not easily separated from the present material, as only stems and not genetic individuals were registered. However, this could be done with relatively high confidence in the field, and therefore we recommend identifying genetic individuals in future long‐term studies of mountain birch.

Such an approach is in accordance with conclusions drawn from research work from Northern Sweden, namely that responses to changes in climate and land use in mountain birch forest lines are slow and lagged in time (Van Bogaert et al., 2011).

Earlier changes in climate and land use could have caused generally increasing genet age throughout the study period. After the development of new agricultural technologies and emigrations to America during the mid‐ to late‐19th century, the exploitation of the outfield pastures became less intense in Norway (Nedkvitne et al., 1995). Local government records show that this was also the case for Hirkjølen after 1845 and coincided with the prohibition of outfield pasturing with goats (Fosvold, 1984). It is not inconceivable that the more intense grazing pressure prior to the 1930s might have hampered establishment and growth and hence affected the age of birch genets in the study period. In this context also the Little Ice Age should be mentioned, which is usually defined as the period between 1300 and 1900, AD with the lowest level of temperatures in the period c.1600 to c.1750 (Gouirand et al., 2008; Linderholm & Gunnarson, 2005; Ljungqvist, 2010; Moberg et al., 2005; Wanner et al., 2008). This cold period is considered to have affected natural dispersal processes and vegetation patterns at high altitudes up until the present time (Kammer et al., 2007; Karlsson et al., 2007; Kullman & Oberg, 2009).

## 5 CONCLUSION

Our study shows that pronounced changes have taken place in the mountain birch protection belt at Hirkjølen in terms of increased biomass and dominant height but also an elevated forest line throughout the last 80 years.

The high survival and slight increase in total biomass in the period 1931–1953 should be seen in connection with the relatively warm summers in the 1930s and 1940s. The warm summers in the period might also have promoted spruce seedling establishment.

This study has revealed a striking high turnover in natural stem dynamics of mountain birch and a great capacity to recover rapidly after a disturbance like moth attacks. From the 1950s and onward, we observe a long‐term trend of increasing biomass and dominant height. Such a trend is difficult to explain as a rapid response to observed changes in growth conditions in terms of climate and land use within the study period. Our findings indicate that parallel to the ability of rapid recovery after disturbance, mountain birch also shows slow, long‐term responses lagged in time to changes in growth conditions. We regard this response as the main driver for the advanced mountain birch forest line by 0.71 m year^−1^. We suggest that the coexistence of these two contradictory features may be understood by relating the rapid‐recover feature to dynamics on the module level, and relating the slow, long‐term response feature to dynamics on the genet level. Our findings stress the need for future long‐term studies of mountain birch to consider the dynamics on both levels. This should be considered when designing refined field methods.

## CONFLICT OF INTEREST

The authors do not have any conflict of interest.

## Supporting information


Appendix S1

Appendix S2

## Data Availability

The data that support the findings of this study are openly deposited in the databank of long‐term experiments at NIBIO and will be transferred to BIRD https://bird.unit.no/.
