# Supplementary figures and images for: Long‐term spatiotemporal dynamics in a mountain birch (Betula pubescens ssp. czerepanovii) forest in south‐east Norway
Source: Plant Environ Interact. 2022 Aug 6;3(4):155–69. doi: 10.1002/pei3.10087 (PMC10168091; doi:10.1002/pei3.10087)

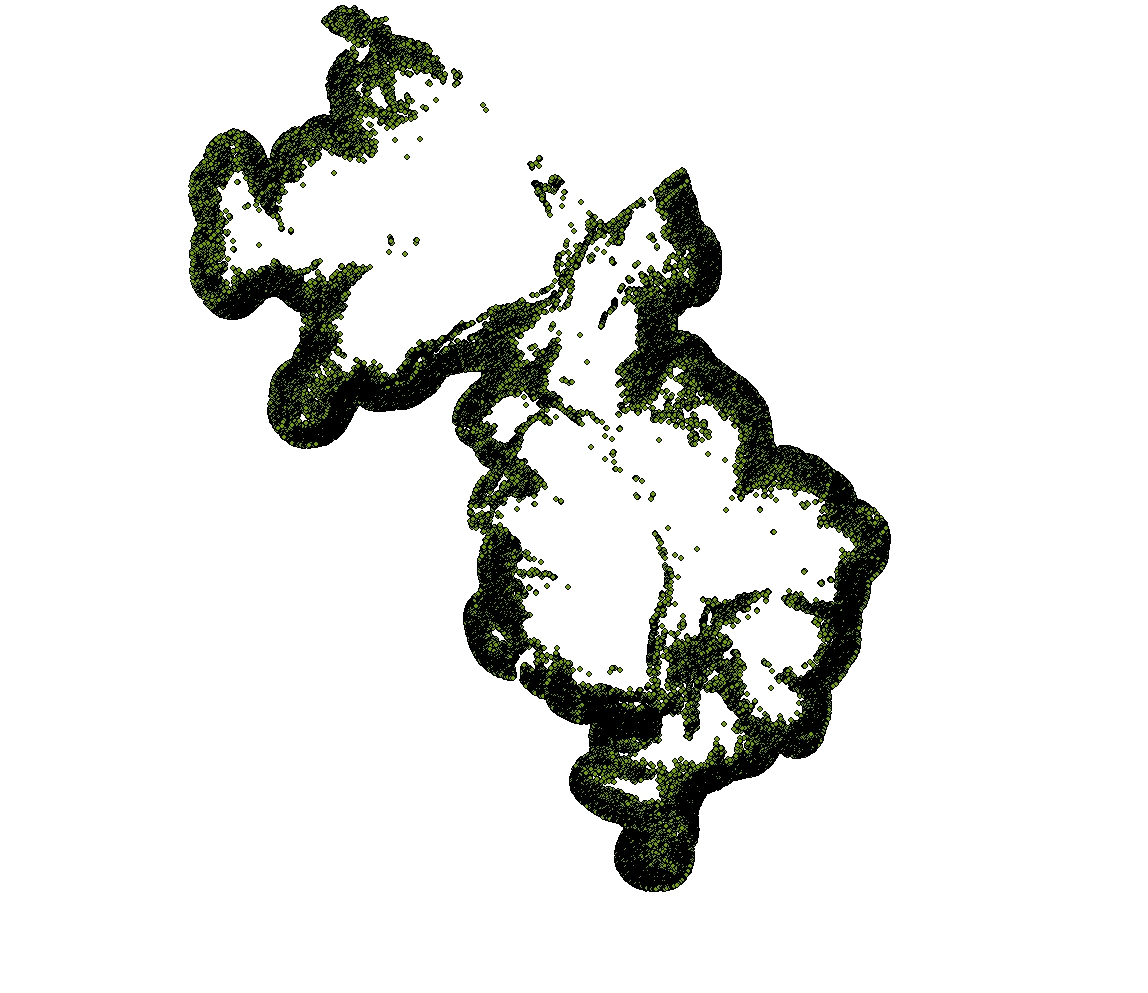

Supplement: Supplementary file 1 — Appendix S1 [file PEI3-3-155-s001.tif]
