# Supplementary material for: Long‐term spatiotemporal dynamics in a mountain birch (Betula pubescens ssp. czerepanovii) forest in south‐east Norway
Source: Plant Environ Interact. 2022 Aug 6;3(4):155–69. doi: 10.1002/pei3.10087 (PMC10168091; doi:10.1002/pei3.10087)
Supplement: Supplementary file 2 — Appendix S2 [file PEI3-3-155-s002.docx]

Supplementary material

Weather observations from the nearest metrological station (Kjøremsgrendi) and local stations within the Hirkjølen research area.

The method for the “down scaling” is described by ([Ojansuu and Henttonen 1983](#_ENREF_43)). Formulas for weight as a function of distance between stations and the plot location, and polynomial regression of elevation, latitude, and longitude are shown beneath.

|  |  |  |  |  |
| --- | --- | --- | --- | --- |
|  |  |  |  |  |
| \| year \| 10Yr mean T \| Triterm 10 yr mean \| 10Yr mean T \| Triterm, 10Yr mean T \| Yr mean T \| Triterm \| Yr mean T \| Triterm \| Triterm \| Triterm \| \| --- \| --- \| --- \| --- \| --- \| --- \| --- \| --- \| --- \| --- \| --- \| \| year \| Interpolert \| interpolert \| Kjøremsgrendi \| Kjøremsgrendi \| Interpolert \| Interpolert \|  \| Kjøremsgrendi \| Furumoen (Mork) \| Alpinstasjonen (Mork) \| \| 1865 \|  \|  \|  \|  \|  \|  \| 0,584616074 \| 9,566665649 \|  \|  \| \| 1866 \|  \|  \|  \|  \|  \|  \| 0,692307637 \| 11,23332977 \|  \|  \| \| 1867 \|  \|  \|  \|  \|  \|  \| -0,784614508 \| 9,63333257 \|  \|  \| \| 1868 \|  \|  \|  \|  \|  \|  \| 1,353845725 \| 12,36666361 \|  \|  \| \| 1869 \|  \|  \|  \|  \|  \|  \| -0,169230415 \| 9,399997711 \|  \|  \| \| 1870 \|  \|  \|  \|  \|  \|  \| 0,961538682 \| 11,59999847 \|  \|  \| \| 1871 \|  \|  \|  \|  \|  \|  \| -0,292307909 \| 11,1333313 \|  \|  \| \| 1872 \|  \|  \|  \|  \|  \|  \| 1,700000066 \| 11,99999746 \|  \|  \| \| 1873 \|  \|  \|  \|  \|  \|  \| 1,684615227 \| 11,36666361 \|  \|  \| \| 1874 \|  \|  \|  \|  \|  \|  \| 0,669230003 \| 9,499994914 \|  \|  \| \| 1875 \|  \|  \| 0,549230747 \| 10,9366638 \|  \|  \| -0,323077037 \| 11,13332876 \|  \|  \| \| 1876 \|  \|  \| 0,500769162 \| 11,0333305 \|  \|  \| 0,207691789 \| 12,19999695 \|  \|  \| \| 1877 \|  \|  \| 0,542307533 \| 11,0866637 \|  \|  \| -0,369230802 \| 10,16666412 \|  \|  \| \| 1878 \|  \|  \| 0,544615272 \| 10,9966638 \|  \|  \| 1,376923121 \| 11,46666463 \|  \|  \| \| 1879 \|  \|  \| 0,612307504 \| 11,2699969 \|  \|  \| 0,507691897 \| 12,13332876 \|  \|  \| \| 1880 \|  \|  \| 0,679230542 \| 11,3466634 \|  \|  \| 1,630769069 \| 12,36666361 \|  \|  \| \| 1881 \|  \|  \| 0,69999989 \| 11,2599968 \|  \|  \| -0,084614428 \| 10,26666514 \|  \|  \| \| 1882 \|  \|  \| 0,705384402 \| 11,25333 \|  \|  \| 1,753845178 \| 11,93332926 \|  \|  \| \| 1883 \|  \|  \| 0,7053844 \| 11,2799967 \|  \|  \| 1,684615209 \| 11,6333313 \|  \|  \| \| 1884 \|  \|  \| 0,828461299 \| 11,5066635 \|  \|  \| 1,899998995 \| 11,7666626 \|  \|  \| \| 1885 \|  \|  \| 0,926922815 \| 11,3633303 \|  \|  \| 0,661538124 \| 9,699996948 \|  \|  \| \| 1886 \|  \|  \| 0,96692286 \| 11,1999969 \|  \|  \| 0,607692242 \| 10,56666311 \|  \|  \| \| 1887 \|  \|  \| 1,126153637 \| 11,209997 \|  \|  \| 1,223076967 \| 10,26666514 \|  \|  \| \| 1888 \|  \|  \| 0,957692027 \| 11,1133301 \|  \|  \| -0,307692986 \| 10,49999491 \|  \|  \| \| 1889 \|  \|  \| 1,080768992 \| 11,0833303 \|  \|  \| 1,738461549 \| 11,83333079 \|  \|  \| \| 1890 \|  \|  \| 1,083076667 \| 10,8233304 \|  \|  \| 1,653845819 \| 9,766665141 \|  \|  \| \| 1891 \|  \|  \| 1,24230727 \| 10,9299967 \|  \|  \| 1,507691603 \| 11,33332825 \|  \|  \| \| 1892 \|  \|  \| 1,059999601 \| 10,7066633 \|  \|  \| -0,069231515 \| 9,699994405 \|  \|  \| \| 1893 \|  \|  \| 0,97538419 \| 10,6633298 \|  \|  \| 0,838461106 \| 11,19999695 \|  \|  \| \| 1894 \|  \|  \| 1,010768834 \| 10,6933299 \|  \|  \| 2,253845435 \| 12,06666311 \|  \|  \| \| 1895 \|  \|  \| 0,984615015 \| 10,8133298 \|  \|  \| 0,39999993 \| 10,89999644 \|  \|  \| \| 1896 \|  \|  \| 1,125384236 \| 10,95333 \| -1,14166667 \| 9,5 \| 2,015384454 \| 11,96666463 \|  \|  \| \| 1897 \|  \|  \| 1,165384139 \| 11,1899963 \| -0,63333333 \| 10,6 \| 1,623075999 \| 12,63332876 \|  \|  \| \| 1898 \|  \|  \| 1,348461048 \| 11,176663 \| -0,675 \| 8,466666667 \| 1,523076094 \| 10,36666107 \|  \|  \| \| 1899 \|  \|  \| 1,257691791 \| 11,1699964 \| -1,09166667 \| 10,2 \| 0,830768989 \| 11,76666514 \|  \|  \| \| 1900 \|  \|  \| 1,133845656 \| 11,3733297 \| -1,575 \| 10,13333333 \| 0,415384467 \| 11,79999797 \|  \|  \| \| 1901 \|  \|  \| 1,149999513 \| 11,6099965 \| -0,4 \| 11,96666667 \| 1,669230168 \| 13,69999695 \|  \|  \| \| 1902 \|  \|  \| 1,162307233 \| 11,5666634 \| -2,04166667 \| 7,933333333 \| 0,053845685 \| 9,266662598 \|  \|  \| \| 1903 \|  \|  \| 1,194614898 \| 11,4499964 \| -0,61666667 \| 9,1 \| 1,161537757 \| 10,03332774 \|  \|  \| \| 1904 \|  \|  \| 1,032307214 \| 11,3133298 \| -1,11666667 \| 9,766666667 \| 0,630768593 \| 10,69999695 \|  \|  \| \| 1905 \| -1,005 \| 9,793333333 \| 1,100768711 \| 11,3733299 \| -0,75833333 \| 10,26666667 \| 1,0846149 \| 11,49999746 \|  \|  \| \| 1906 \| -0,834166667 \| 9,89 \| 1,06999952 \| 11,2833298 \| 0,56666667 \| 10,46666667 \| 1,70769255 \| 11,06666311 \|  \|  \| \| 1907 \| -0,8 \| 9,666666667 \| 1,02461501 \| 10,9599968 \| -0,29166667 \| 8,366666667 \| 1,169230901 \| 9,399998983 \|  \|  \| \| 1908 \| -0,725833333 \| 9,83 \| 1,034615095 \| 11,0499972 \| 0,06666667 \| 10,1 \| 1,623076943 \| 11,26666514 \|  \|  \| \| 1909 \| -0,703333333 \| 9,73 \| 0,992307423 \| 10,8999969 \| -0,86666667 \| 9,2 \| 0,407692267 \| 10,2666626 \|  \|  \| \| 1910 \| -0,524166667 \| 9,736666667 \| 1,13769207 \| 10,9199969 \| 0,21666667 \| 10,2 \| 1,869230931 \| 11,99999746 \|  \|  \| \| 1911 \| -0,4375 \| 9,543333333 \| 1,14461512 \| 10,6799967 \| 0,46666667 \| 10,03333333 \| 1,738460669 \| 11,29999542 \|  \|  \| \| 1912 \| -0,251666667 \| 9,846666667 \| 1,289999805 \| 10,9933304 \| -0,18333333 \| 10,96666667 \| 1,507692539 \| 12,39999898 \|  \|  \| \| 1913 \| -0,135833333 \| 9,976666667 \| 1,383076724 \| 11,1133306 \| 0,54166667 \| 10,4 \| 2,092306944 \| 11,23332977 \|  \|  \| \| 1914 \| 0,070833333 \| 10,21666667 \| 1,555384365 \| 11,359997 \| 0,95 \| 12,16666667 \| 2,353845009 \| 13,16666158 \|  \|  \| \| 1915 \| -0,015833333 \| 10,04 \| 1,417692059 \| 11,2133303 \| -1,625 \| 8,5 \| -0,292308165 \| 10,03333028 \|  \|  \| \| 1916 \| -0,1225 \| 9,923333333 \| 1,379999758 \| 11,206664 \| -0,5 \| 9,3 \| 1,330769539 \| 11 \|  \|  \| \| 1917 \| -0,225 \| 10,17 \| 1,331538218 \| 11,4899974 \| -1,31666667 \| 10,83333333 \| 0,684615502 \| 12,23333232 \|  \|  \| \| 1918 \| -0,274166667 \| 10,08333333 \| 1,31769199 \| 11,4233304 \| -0,425 \| 9,233333333 \| 1,484614666 \| 10,59999593 \|  \|  \| \| 1919 \| -0,320833333 \| 10,13 \| 1,333076596 \| 11,4833305 \| -1,33333333 \| 9,666666667 \| 0,561538329 \| 10,86666361 \|  \|  \| \| 1920 \| -0,366666667 \| 9,98 \| 1,369230367 \| 11,3166639 \| -0,24166667 \| 8,7 \| 2,230768639 \| 10,33333079 \|  \|  \| \| 1921 \| -0,4325 \| 9,833333333 \| 1,380768908 \| 11,1333309 \| -0,19166667 \| 8,566666667 \| 1,853846073 \| 9,466665904 \|  \|  \| \| 1922 \| -0,54 \| 9,623333333 \| 1,312307303 \| 10,896664 \| -1,25833333 \| 8,866666667 \| 0,823076496 \| 10,03333028 \|  \|  \| \| 1923 \| -0,7625 \| 9,383333333 \| 1,121538145 \| 10,6933309 \| -1,68333333 \| 8 \| 0,184615364 \| 9,19999822 \|  \|  \| \| 1924 \| -0,914166667 \| 9,096666667 \| 1,045384361 \| 10,4499977 \| -0,56666667 \| 9,3 \| 1,592307164 \| 10,73332977 \|  \|  \| \| 1925 \| -0,825833333 \| 9,36 \| 1,209999772 \| 10,6933311 \| -0,74166667 \| 11,13333333 \| 1,353845945 \| 12,46666463 \|  \|  \| \| 1926 \| -0,860833333 \| 9,463333333 \| 1,216153487 \| 10,776664 \| -0,85 \| 10,33333333 \| 1,392306695 \| 11,83332825 \|  \|  \| \| 1927 \| -0,894166667 \| 9,363333333 \| 1,204614996 \| 10,6799971 \| -1,65 \| 9,833333333 \| 0,569230593 \| 11,26666387 \|  \|  \| \| 1928 \| -1,014166667 \| 9,183333333 \| 1,134615115 \| 10,5099974 \| -1,625 \| 7,433333333 \| 0,784615847 \| 8,899998983 \|  \|  \| \| 1929 \| -0,985833333 \| 9,033333333 \| 1,184615107 \| 10,3799974 \| -1,05 \| 8,166666667 \| 1,061538256 \| 9,566663106 \|  \|  \| \| 1930 \| -0,884166667 \| 9,27 \| 1,279999735 \| 10,6266641 \| 0,775 \| 11,06666667 \| 3,184614915 \| 12,79999797 \|  \|  \| \| 1931 \| -1,005 \| 9,29 \| 1,179999718 \| 10,706664 \| -1,4 \| 8,766666667 \| 0,853845908 \| 10,26666514 \|  \|  \| \| 1932 \| -0,835833333 \| 9,416666667 \| 1,325384298 \| 10,826664 \| 0,43333333 \| 10,13333333 \| 2,27692229 \| 11,23332977 \| 10,2333333 \|  \| \| 1933 \| -0,630833333 \| 9,763333333 \| 1,539999653 \| 11,2066639 \| 0,36666667 \| 11,46666667 \| 2,33076892 \| 12,99999746 \| 12,0333333 \|  \| \| 1934 \| -0,478333333 \| 9,916666667 \| 1,693845783 \| 11,346664 \| 0,95833333 \| 10,83333333 \| 3,130768464 \| 12,1333313 \| 11,1333333 \|  \| \| 1935 \| -0,446666667 \| 9,856666667 \| 1,72230728 \| 11,2733306 \| -0,425 \| 10,53333333 \| 1,638460911 \| 11,73332977 \| 10,6 \|  \| \| 1936 \| -0,363333333 \| 9,946666667 \| 1,770768897 \| 11,3199975 \| -0,01666667 \| 11,23333333 \| 1,876922869 \| 12,29999797 \| 11,4666667 \|  \| \| 1937 \| -0,181666667 \| 10,17666667 \| 1,903076552 \| 11,5233309 \| 0,16666667 \| 12,13333333 \| 1,892307135 \| 13,29999797 \| 11,9333333 \| 11,5333333 \| \| 1938 \| 0,118333333 \| 10,47 \| 2,132307173 \| 11,7866641 \| 1,375 \| 10,36666667 \| 3,076922059 \| 11,53333028 \| 10,8333333 \| 10,2666667 \| \| 1939 \| 0,22 \| 10,69333333 \| 2,203845602 \| 12,0233307 \| -0,03333333 \| 10,4 \| 1,776922551 \| 11,93332926 \| 11,3666667 \| 10,0333333 \| \| 1940 \| -0,046666667 \| 10,55 \| 1,907691797 \| 11,8299973 \| -1,89166667 \| 9,633333333 \| 0,223076866 \| 10,86666361 \| 10,2666667 \| 8,36666667 \| \| 1941 \| -0,114166667 \| 10,71333333 \| 1,849230208 \| 12,009997 \| -2,075 \| 10,4 \| 0,269230017 \| 12,06666311 \| 10,9 \| 9,76666667 \| \| 1942 \| -0,336666667 \| 10,58333333 \| 1,641537983 \| 11,9266637 \| -1,79166667 \| 8,833333333 \| 0,200000039 \| 10,39999644 \| 9,43333333 \|  \| \| 1943 \| -0,320833333 \| 10,38666667 \| 1,638461033 \| 11,729997 \| 0,525 \| 9,5 \| 2,29999942 \| 11,03333028 \| 10,1333333 \|  \| \| 1944 \| -0,426666667 \| 10,29333333 \| 1,514614948 \| 11,6733304 \| -0,1 \| 9,9 \| 1,892307612 \| 11,56666565 \| 10,7333333 \|  \| \| 1945 \| -0,359166667 \| 10,35333333 \| 1,566153396 \| 11,7699972 \| 0,25 \| 11,13333333 \| 2,153845388 \| 12,69999695 \| 11,3666667 \|  \| \| 1946 \| -0,358333333 \| 10,19333333 \| 1,566153384 \| 11,6933304 \| -0,00833333 \| 9,633333333 \| 1,876922754 \| 11,53333028 \| 10,3666667 \|  \| \| 1947 \| -0,45 \| 10,22333333 \| 1,473845752 \| 11,7333303 \| -0,75 \| 12,43333333 \| 0,969230817 \| 13,69999695 \| 11,7666667 \|  \| \| 1948 \| -0,54 \| 10,14333333 \| 1,381538069 \| 11,7133301 \| 0,475 \| 9,566666667 \| 2,153845223 \| 11,33332825 \| 9,7 \| 7,86666667 \| \| 1949 \| -0,458333333 \| 10,02666667 \| 1,487691906 \| 11,6333303 \| 0,78333333 \| 9,233333333 \| 2,838460922 \| 11,1333313 \| 9,33333333 \| 8,3 \| \| 1950 \| -0,329166667 \| 10,01666667 \| 1,619230329 \| 11,6866636 \| -0,6 \| 9,533333333 \| 1,538461098 \| 11,39999644 \| 10,2333333 \| 8,96666667 \| \| 1951 \| -0,185833333 \| 9,873333333 \| 1,722307229 \| 11,5266635 \| -0,64166667 \| 8,966666667 \| 1,299999017 \| 10,46666209 \| 9,66666667 \| 8,26666667 \| \| 1952 \| -0,1525 \| 9,793333333 \| 1,750768737 \| 11,4733302 \| -1,45833333 \| 8,033333333 \| 0,484615115 \| 9,866663615 \| 8,9 \| 7 \| \| 1953 \| -0,0725 \| 9,933333333 \| 1,832307221 \| 11,629997 \| 1,325 \| 10,9 \| 3,115384267 \| 12,59999847 \| 11,4666667 \| 9,9 \| \| 1954 \| -0,145 \| 9,86 \| 1,756153322 \| 11,5699969 \| -0,825 \| 9,166666667 \| 1,13076862 \| 10,96666463 \| 9,76666667 \| 8,33333333 \| \| 1955 \| -0,219166667 \| 9,87 \| 1,671537985 \| 11,5766635 \| -0,49166667 \| 11,23333333 \| 1,307692014 \| 12,7666626 \| 11,6666667 \| 10,6666667 \| \| 1956 \| -0,3325 \| 9,7 \| 1,580768804 \| 11,4133303 \| -1,14166667 \| 7,933333333 \| 0,969230945 \| 9,899998983 \| 9 \| 7,26666667 \| \| 1957 \| -0,29 \| 9,346666667 \| 1,657691835 \| 11,1066635 \| -0,325 \| 8,9 \| 1,738461128 \| 10,63332876 \| 9,56666667 \| 8,3 \| \| 1958 \| -0,486666667 \| 9,293333333 \| 1,511538082 \| 11,076664 \| -1,49166667 \| 9,033333333 \| 0,692307692 \| 11,03333282 \| 9,83333333 \| 8,36666667 \| \| 1959 \| -0,495 \| 9,45 \| 1,508461165 \| 11,1966639 \| 0,7 \| 10,8 \| 2,807691757 \| 12,33333079 \| 11,6 \| 10,2666667 \| \| 1960 \| -0,543333333 \| 9,473333333 \| 1,47384577 \| 11,2333305 \| -1,08333333 \| 9,766666667 \| 1,192307142 \| 11,7666626 \| 10,5 \| 9,2 \| \| 1961 \| -0,456666667 \| 9,493333333 \| 1,589999613 \| 11,2699974 \| 0,225 \| 9,166666667 \| 2,461537453 \| 10,83333079 \| 9,73333333 \| 8,4 \| \| 1962 \| -0,475833333 \| 9,43 \| 1,621538052 \| 11,2299975 \| -1,65 \| 7,4 \| 0,799999503 \| 9,466664632 \| 8,1 \| 6,5 \| \| 1963 \| -0,6925 \| 9,336666667 \| 1,481538089 \| 11,1799975 \| -0,84166667 \| 9,966666667 \| 1,71538463 \| 12,09999847 \| 10,5333333 \| 9,36666667 \| \| 1964 \| -0,651666667 \| 9,223333333 \| 1,562307357 \| 11,0666641 \| -0,41666667 \| 8,033333333 \| 1,938461304 \| 9,83333079 \| 9,1 \| 7,4 \| \| 1965 \| -0,738333333 \| 8,936666667 \| 1,50384583 \| 10,8299975 \| -1,35833333 \| 8,366666667 \| 0,723076747 \| 10,39999644 \| 9,33333333 \| 7,9 \| \| 1966 \| -0,835833333 \| 9,166666667 \| 1,426922783 \| 11,0466639 \| -2,11666667 \| 10,23333333 \| 0,200000479 \| 12,06666311 \| 11,1 \| 9,76666667 \| \| 1967 \| -0,850833333 \| 9,206666667 \| 1,419999662 \| 11,0633306 \| -0,475 \| 9,3 \| 1,669229911 \| 10,79999542 \|  \|  \| \| 1968 \| -0,8225 \| 9,313333333 \| 1,398461107 \| 11,1366636 \| -1,20833333 \| 10,1 \| 0,476922145 \| 11,7666626 \|  \|  \| \| 1969 \| -0,988333333 \| 9,41 \| 1,190768744 \| 11,25333 \| -0,95833333 \| 11,76666667 \| 0,73076813 \| 13,49999491 \|  \|  \| \| 1970 \| -1,014166667 \| 9,466666667 \| 1,139999529 \| 11,3166634 \| -1,34166667 \| 10,33333333 \| 0,684614988 \| 12,39999644 \|  \|  \| \| 1971 \| -1,050833333 \| 9,41 \| 1,080768841 \| 11,2999967 \| -0,14166667 \| 8,6 \| 1,869230573 \| 10,66666412 \|  \|  \| \| 1972 \| -0,895 \| 9,653333333 \| 1,15243548 \| 11,5299965 \| -0,09166667 \| 9,833333333 \| 1,516665896 \| 11,7666626 \|  \|  \| \| 1973 \| -0,8025 \| 9,653333333 \| 1,168589288 \| 11,4633298 \| 0,08333333 \| 9,966666667 \| 1,876922708 \| 11,43333181 \|  \|  \| \| 1974 \| -0,729166667 \| 9,686666667 \| 1,19782003 \| 11,5166631 \| 0,31666667 \| 8,366666667 \| 2,230768726 \| 10,36666361 \|  \|  \| \| 1975 \| -0,5225 \| 9,863333333 \| 1,390127686 \| 11,6633298 \| 0,70833333 \| 10,13333333 \| 2,646153303 \| 11,86666361 \|  \|  \| \| 1976 \| -0,4075 \| 9,836666667 \| 1,450960906 \| 11,6299965 \| -0,96666667 \| 9,966666667 \| 0,808332682 \| 11,73332977 \|  \|  \| \| 1977 \| -0,469166667 \| 9,76 \| 1,423204572 \| 11,6433299 \| -1,09166667 \| 8,533333333 \| 1,391666571 \| 10,93332926 \|  \|  \| \| 1978 \| -0,495 \| 9,656666667 \| 1,419678982 \| 11,5399966 \| -1,46666667 \| 9,066666667 \| 0,441666245 \| 10,73332977 \|  \|  \| \| 1979 \| -0,565833333 \| 9,37 \| 1,348140651 \| 11,2666634 \| -1,66666667 \| 8,9 \| 0,015384821 \| 10,7666626 \|  \|  \| \| 1980 \| -0,53 \| 9,356666667 \| 1,367371443 \| 11,2466634 \| -0,98333333 \| 10,2 \| 0,876922901 \| 12,19999695 \|  \|  \| \| 1981 \| -0,650833333 \| 9,366666667 \| 1,22890984 \| 11,2099965 \| -1,35 \| 8,7 \| 0,484614546 \| 10,29999542 \|  \|  \| \| 1982 \| -0,656666667 \| 9,34 \| 1,268781658 \| 11,1766632 \| -0,15 \| 9,566666667 \| 1,915384073 \| 11,43332926 \|  \|  \| \| 1983 \| -0,6325 \| 9,35 \| 1,310320015 \| 11,1499962 \| 0,325 \| 10,06666667 \| 2,292306276 \| 11,16666158 \|  \|  \| \| 1984 \| -0,675833333 \| 9,45 \| 1,280320024 \| 11,226663 \| -0,11666667 \| 9,366666667 \| 1,93076882 \| 11,1333313 \|  \|  \| \| 1985 \| -0,921666667 \| 9,38 \| 1,048012295 \| 11,1566628 \| -1,75 \| 9,433333333 \| 0,32307601 \| 11,16666158 \|  \|  \| \| 1986 \| -0,9175 \| 9,346666667 \| 1,079486639 \| 11,1233294 \| -0,925 \| 9,633333333 \| 1,123076127 \| 11,39999644 \|  \|  \| \| 1987 \| -0,9675 \| 9,286666667 \| 1,003396819 \| 11,0133293 \| -1,59166667 \| 7,933333333 \| 0,630768372 \| 9,833328247 \|  \|  \| \| 1988 \| -0,81 \| 9,476666667 \| 1,187691706 \| 11,2033295 \| 0,10833333 \| 10,96666667 \| 2,284615113 \| 12,6333313 \|  \|  \| \| 1989 \| -0,555 \| 9,53 \| 1,493076214 \| 11,1999962 \| 0,88333333 \| 9,433333333 \| 3,069229896 \| 10,73332977 \|  \|  \| \| 1990 \| -0,339166667 \| 9,486666667 \| 1,736922293 \| 11,1466629 \| 1,175 \| 9,766666667 \| 3,3153837 \| 11,66666412 \|  \|  \| \| 1991 \| -0,180833333 \| 9,6 \| 1,915383864 \| 11,2799965 \| 0,23333333 \| 9,833333333 \| 2,269230256 \| 11,6333313 \|  \|  \| \| 1992 \| -0,115833333 \| 9,6 \| 1,980768457 \| 11,2399966 \| 0,5 \| 9,566666667 \| 2,569229997 \| 11,03333028 \|  \|  \| \| 1993 \| -0,2225 \| 9,356666667 \| 1,887691711 \| 11,0666636 \| -0,74166667 \| 7,633333333 \| 1,361538814 \| 9,433331807 \|  \|  \| \| 1994 \| -0,274166667 \| 9,453333333 \| 1,848460931 \| 11,1399968 \| -0,63333333 \| 10,33333333 \| 1,538461025 \| 11,86666361 \|  \|  \| \| 1995 \| -0,1375 \| 9,52 \| 1,988460935 \| 11,1566635 \| -0,38333333 \| 10,1 \| 1,72307605 \| 11,33332825 \|  \|  \| \| 1996 \| -0,166666667 \| 9,546666667 \| 1,956922571 \| 11,1699969 \| -1,21666667 \| 9,9 \| 0,807692491 \| 11,53333028 \|  \|  \| \| 1997 \| 0,033333333 \| 9,97 \| 2,149999502 \| 11,5566638 \| 0,40833333 \| 12,16666667 \| 2,561537678 \| 13,69999695 \|  \|  \| \| 1998 \| -1,94289E-17 \| 9,696666667 \| 2,107371235 \| 11,2933304 \| -0,225 \| 8,233333333 \| 1,85833244 \| 9,999997457 \|  \|  \| \| 1999 \| -0,066666667 \| 9,723333333 \| 2,057114774 \| 11,359997 \| 0,21666667 \| 9,7 \| 2,566665292 \| 11,39999644 \|  \|  \| \| 2000 \| -0,081666667 \| 9,633333333 \| 2,050576317 \| 11,229997 \| 1,025 \| 8,866666667 \| 3,249999126 \| 10,36666361 \|  \|  \| \| 2001 \| -0,160833333 \| 9,616666667 \| 1,989486518 \| 11,1799967 \| -0,55833333 \| 9,666666667 \| 1,658332268 \| 11,13332876 \|  \|  \| \| 2002 \| -0,176666667 \| 9,843333333 \| 1,993396814 \| 11,4199969 \| 0,34166667 \| 11,83333333 \| 2,608332952 \| 13,43333181 \|  \|  \| \| 2003 \| -0,034166667 \| 10,17 \| 2,153909563 \| 11,7666634 \| 0,68333333 \| 10,9 \| 2,966666311 \| 12,89999644 \|  \|  \| \| 2004 \| 0,091666667 \| 10,09666667 \| 2,279230088 \| 11,73333 \| 0,625 \| 9,6 \| 2,791666269 \| 11,53333028 \|  \|  \| \| 2005 \| 0,206666667 \| 10,06 \| 2,421088974 \| 11,7499967 \| 0,76666667 \| 9,733333333 \| 3,141664912 \| 11,49999491 \|  \|  \| \| 2006 \| 0,47 \| 10,27 \| 2,702819672 \| 11,9633301 \| 1,41666667 \| 12 \| 3,624999473 \| 13,66666412 \|  \|  \| \| 2007 \| 0,5075 \| 10,11666667 \| 2,714165882 \| 11,8133303 \| 0,78333333 \| 10,63333333 \| 2,674999774 \| 12,19999949 \|  \|  \| \| 2008 \|  \| 10,30333333 \|  \|  \|  \| 10,1 \|  \|  \|  \|  \| |  |  |  |  |
